# Non-Invasive In Vivo Estimation of HbA1c Using Monte Carlo Photon Propagation Simulation: Application of Tissue-Segmented 3D MRI Stacks of the Fingertip and Wrist for Wearable Systems

**DOI:** 10.3390/s23010540

**Published:** 2023-01-03

**Authors:** Shifat Hossain, Ki-Doo Kim

**Affiliations:** 1 Department of Electrical and Computer Engineering, University of Central Florida, Orlando, FL 32816, USA; 2 Department of Electronics Engineering, Kookmin University, Seoul 02707, Republic of Korea

**Keywords:** HbA1c, noninvasive, in vivo, Monte Carlo, tissue segmentation, MRI

## Abstract

The early diagnosis of diabetes mellitus in normal people or maintaining stable blood sugar concentrations in diabetic patients requires frequent monitoring of the blood sugar levels. However, regular monitoring of the sugar levels is problematic owing to the pain and inconvenience associated with pricking the fingertip or using minimally invasive patches. In this study, we devise a noninvasive method to estimate the percentage of the in vivo glycated hemoglobin (HbA1c) values from Monte Carlo photon propagation simulations, based on models of the wrist using 3D magnetic resonance (MR) image data. The MR image slices are first segmented for several different tissue types, and the proposed Monte Carlo photon propagation system with complex composite tissue support is then used to derive several models for the fingertip and wrist sections with different wavelengths of light sources and photodetector arrangements. The Pearson r values for the estimated percent HbA1c values are 0.94 and 0.96 for the fingertip transmission- and reflection-type measurements, respectively. This is found to be the best among the related studies. Furthermore, a single-detector multiple-source arrangement resulted in a Pearson r value of 0.97 for the wrist. The Bland–Altman bias values were found to be −0.003 ± 0.36, 0.01 ± 0.25, and 0.01 ± 0.21, for the two fingertip and wrist models, respectively, which conform to the standards of the current state-of-the-art invasive point-of-care devices. The implementation of these algorithms will be a suitable alternative to the invasive state-of-the-art methods.

## 1. Introduction

Diabetes mellitus is a metabolic disorder characterized by the presence of high concentrations of sugar in the bloodstream. In addition to high blood sugar levels, elevated thirst and appetite, and frequent urination are the common symptoms of diabetes, which may cause serious long-term complications if left untreated; diabetes is often a result of disorders related to the insulin production or the cellular responses to insulin molecules. Type I diabetes occurs mostly because the pancreas fails to produce insulin molecules. Moreover, type II diabetes is caused by the abnormal responses of the bodily cells to insulin. Although the ingestion of foods with large amounts of sugar is not directly related to the cause of diabetes, excess weight and a sedentary lifestyle are two of the major causes of type II diabetes. Blood glucose levels can be directly estimated by measuring the amount of glucose in the bloodstream, using a variety of invasive and noninvasive methods, based on chemical, electrochemical, or optical processes [1,2,3,4]. Moreover, blood glucose levels can also be estimated by analyzing the glycated hemoglobin (HbA1c) component in the blood. The higher the amount of glycated hemoglobin present in the blood, the higher the probability for the person to be diabetic.

Glycated hemoglobin (HbA1c) is a form of hemoglobin that is non-enzymatically bonded with saccharides. In general, most monosaccharides spontaneously form bonds with the hemoglobin compounds, resulting in glycated hemoglobin molecules. Once a bond is formed, the glycated molecules do not degrade to debond from the saccharides until the end of the lifespan of the red blood cells (RBCs). Hence, the HbA1c level in the blood is considered to be directly proportional to the weighted average of the blood glucose levels over a 4-month period (typical lifespan of the RBCs). For the same reason, the measurement of HbA1c is considered an important marker for diagnosing diabetes.

There are several invasive processes available for measuring the HbA1c levels from blood samples. Among them, ion-exchange high-performance liquid chromatography (HPLC), enzymatic assays, and boronate affinity chromatography are the most common methods [5]. However, there are very few studies on noninvasive measurement procedures to date. One reported study focused on the development of an in vitro HbA1c measurement procedure using optical sensors [6]; however, this approach requires blood samples and involves an invasive process.

Another group of researchers classified mice models into diabetic, obese, and normal control groups, based on the measurement conditions associated with hyperglycemia [7]. Some other studies reported the classification into diabetic and non-diabetic statuses for subjects [8]. The HbA1c levels were also estimated using the acetone levels in the breath in another study [9]. We previously attempted to estimate the HbA1c levels by simple gray-box models derived from Beer–Lambert-based equations, using digital volume pulse waveforms [10]. Although our previous work shows promising results, it can produce erroneous estimations in extreme cases with too low or too high amounts of hematocrit and very low blood oxygenation (SpO_2_) values.

In this work, we developed a method to estimate the percentage of the HbA1c values from the human wrist-anatomy-based models with the consideration of wearable applications. The anatomy references were derived from 3D magnetic resonance (MR) image slices and segmented for several tissue types. Finally, several models were derived from the tissue-segmented anatomy references by a proposed voxel-based Monte Carlo photon propagation system. The proposed Monte Carlo photon propagation system has been shown to implement the complex composite tissue materials with a simple architecture, which can solve the resolution and performance tradeoffs of the voxel-based simulator systems. These derived models were used to estimate the in vivo percentage HbA1c values through the acquired photoplethysmography (PPG) signals at multiple wavelengths. We acquired the PPG signals at three different wavelengths, namely 465 nm, 525 nm, and 615 nm, which are the median wavelengths of a RGB color sensor. Establishing our HbA1c estimation method can greatly improve the use of mobile-camera-based PPG sensors for the accurate estimation of the HbA1c values, thereby resulting in low-cost diagnostic devices.

The contributions of our Monte Carlo photon propagation system to estimate the HbA1c values in-vivo are summarized as follows:

1. We have used a novel composite tissue material model derived from MR images and Monte Carlo simulations with multiple source signals to minimize the estimation errors;
2. We have shown significant improvements in the estimation accuracy of HbA1c as well as SpO_2_ through the comparison with the previously studied methods [10,11] for the fingertip model;
3. The proposed the wrist model with multiple light-emitting diodes (LEDs) and a single PD exhibited the highest correlation with the reference experimental data among the three tested models;
4. Establishing our HbA1c estimation method can greatly improve the use of mobile-camera-based PPG sensors for the accurate estimation of the HbA1c values, thereby resulting in low-cost diagnostic devices.

## 2. Methodology

**Figure 1:**
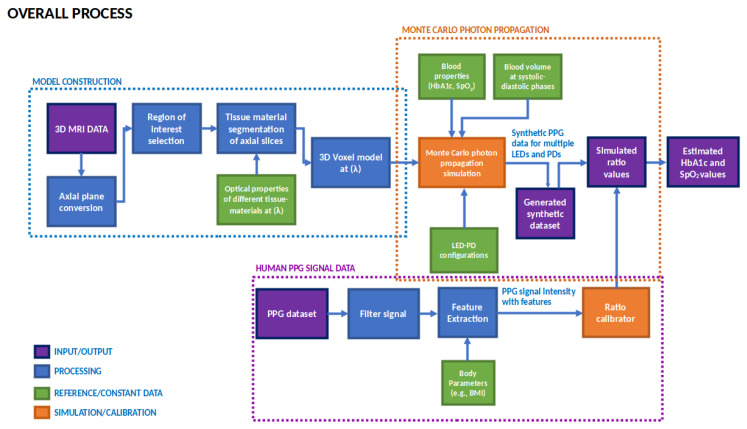
Overall block diagram of the HbA1c estimation method using the Monte Carlo photon propagation simulation.

In this study, the noninvasive method of estimating the HbA1c levels was developed, as noted previously. The entire process initially begins with the design of the Monte Carlo photon propagation simulation scheme using voxels and a composite tissue material supporting algorithm. Then, the 3D models of the fingertip and wrist were constructed by segmenting the MR image slices, depending on the tissue types. The 3D model is then used to run the photon propagation simulation using the custom-designed algorithm. The simulation is run for different physiological conditions (blood properties, systolic and diastolic pulsatile properties, etc.) set in the 3D model, resulting in the intensity data received in the virtual photodetector in the simulation environment. Finally, the 3-dimensional Monte Carlo simulation result data were fed into a calibration model targeting the intensity values of the Monte Carlo simulations, given the input of the PPG signal intensity values with the temporal- and frequency-domain features to produce reliable and accurate estimations of the HbA1c levels. The overall block diagram of the proposed method is shown in Figure 1.

The overall process is segmented into three parts—model construction, Monte Carlo photon propagation simulation, and human PPG signal data processing. The model construction part contains the process of generating the parametric tissue model with the help of MR image data for the Monte Carlo simulation. Then, the Monte Carlo photon propagation simulation part contains the individual components of the simulation blocks. This simulator takes in the parametric model generated in the previous step and runs the photon propagation simulation to generate the optical models which can take the PPG signal as input to estimate the HbA1c values. Then, using the human PPG data, the optical models are evaluated. The preprocessing of the human data was described in the human PPG signal data blocks. The different blocks and elements depicted in this block diagram are discussed in detail in the following subsections.

### 2.1. Monte Carlo Photon Propagation

#### 2.1.1. Photon Propagation Theory

The Monte Carlo methods are a class of algorithms that rely on random sampling to produce numerical results. These methods are often used to solve mathematical and physical problems. Although Monte Carlo-based methods are sometimes computationally intensive, these can be very flexible and are capable of solving problems that no other methods can. Among the different implementations of the Monte Carlo-based methods, the photon propagation in the turbid biological media is crucial in many biomedical imaging applications.

First, we construct a simplified Monte Carlo photon transport mechanism. This process can be described under four main steps, namely the photon packet generation, the packet movement with the dynamic spatial step size, the absorption and scattering of photon packets, and the photon termination. The Monte Carlo photon propagation method is detailed in Algorithm 1.
**Algorithm 1**: Pseudocode of the Monte Carlo photon propagation process1 2 3 4 5 6 7 8 9 10 11 12 13 14 15 16 17 18 19 20 21 22 23 24 25 26 27***Initialize:****system_variables, voxel_model, total_photon_number, photon_data* ***for** total_photon_number:* *photon <= generatePhotonPacket(photon_location, photon_direction)* ***while** photon.weight > 0 and photon.is_out == False:* *tissue_type<= calculateTissueMedium(photon)* *step <= calculateStepSize(tissue_type)* *movePhoton(photon)* ***if** photonCrossedTissueBoundary(photon):* *r <= calculateReflectionCoefficient(photon)* *R <= calculatePowerReflectionCoefficient(r)* *photon <= reflect_refractPhoton(photon, R)* ***endif*** *absorbPhotonWeight(photon)* *recordPhotonData(photon)* *scatterPhoton(photon)* ***if** photon.weight < roulette_cutoff:* *photon <= photonRoulette(photon)* ***endif*** ***endwhile*** ***endfor***

In the above algorithm, each photon packet is generated with a weight of 1 with a specific position and direction. The source is selected as a pencil type that emits from a point source in a predefined direction. Next, the tissue medium type is calculated according to the current position of the photon. The step size is then calculated from the tissue type using the following equation:

(1) $$step=\frac{-ln\xi }{\mu_{t}}$$

where $\mu_{t}$ is the total absorption coefficient of the tissue medium in which the photon is currently residing; ξ is a random number sampled from a uniform distribution of 0 to 1.

The photon packet then moves with its designated step size and direction vector. The position updating equations are given below:

(2) $$x=x+u_{x}\times step$$

(3) $$y=y+u_{y}\times step$$

(4) $$z=z+u_{z}\times step$$

Here, ($u_{x}$, $u_{y}$, $u_{z}$) is the unit direction vector of the photon packet.

Then, the photon packet is evaluated for crossing a tissue boundary. If the photon packet has passed a boundary, its reflection coefficient at the boundary is calculated from Schilick’s approximation of the Fresnel equation. According to Schilick’s model, the reflection coefficient $r\left(\theta \right)$ is calculated, as follows:

(5) $$r\left(\theta \right)=r_{0}+\left(1-r_{0}\right){\left(1-cos\theta \right)}^{5}$$

Here, $r_{0}$ and θ are the reflection coefficient of the photon packet incident normal to the surface and the angle between the surface normal vector and direction vector of the photon packet, respectively. The value of $r_{0}\;$ can be calculated by the following equation:

(6) $$r_{0}={\left(\frac{n_{1}-n_{2}}{n_{1}+n_{2}}\right)}^{2}$$

where $n_{1}$ and $n_{2}$ are the refraction indices of the two consecutive tissue media through which the photon packet travels. From this reflection coefficient, the reflectance R is calculated as:

(7) $$R={\left|r\right|}^{2}$$

Thus, the transmittance is given, as follows:

(8) T=1−R

From these transmittance and reflectance values, the probability of the photon being transmitted or reflected is calculated. If the photon is transmitted, then it follows Snell’s law of refraction for the new position and direction. Moreover, if the photon is reflected in the same medium, the law of reflection is conserved for calculating its new position and direction.

Following the modification of the position and direction vector of the photon packet, the absorption and scattering effects of the photon, due to the tissue medium are calculated. The weight parameter (W) of the photon packet is updated in each absorption step of the simulation. Moreover, the updated weight values are stored along with other information for further analyses (e.g., photon fluence rate, intensity). The weight of the photon packet in a tissue medium is updated as follows:

(9) $$W=W-\Delta W,\;\mathrm{where}\;\Delta W=\frac{\mu_{a}}{\mu_{t}}$$

In Equation (9), $\mu_{a}$ is the absorption coefficient of the blood component. Next, after updating the weight value of the photon packet, the photon direction vector is updated using the Henyey–Greenstein phase function. The scattering angle (θ) is calculated from the phase function formula as:

(10) $$cos\theta =\left(\begin{array}{c} \frac{1}{2g}\left[1+g^{2}-\left(\frac{1-g^{2}}{1-g+2g\xi }\right)\right],\;if\;g\neq 0\\ 1-2\xi ,\;if\;g=0 \end{array}\right.$$

The term g is the scattering anisotropy factor of the tissue medium. Then, the polar angle is also calculated for updating the direction vector of the photon packet, as follows:

(11) ϕ=2πξ

In Equations (10) and (11), ξ is a random number sampled from a uniform distribution from 0 to 1. Finally, the direction vector of the photon packet is updated, as follows:

(12) $${u_{x}}^{\prime }=\frac{sin\theta \left(u_{x}u_{z}cos\phi -u_{y}sin\phi \right)}{\sqrt{1-\mu_{z}^{2}}}+u_{x}cos\theta$$

(13) $${u_{y}}^{\prime }=\frac{sin\theta \left(u_{y}u_{z}cos\phi +u_{x}sin\phi \right)}{\sqrt{1-\mu_{z}^{2}}}+u_{y}cos\theta$$

(14) $${u_{z}}^{\prime }=-\sqrt{1-u_{z}^{2}}sin\theta cos\phi +u_{z}cos\theta$$

Following this stage, if the photon weight is less than the roulette cutoff value (in our case, we designate this value as 0.001), then the weight of the photon packet is updated by the following method to conserve the total energy of the system:

(15) $$W=\left(\begin{array}{c} r_{c}W,\;if\;\xi \leq 1/r_{c}\\ 0,\;if\;\xi >1/r_{c} \end{array}\right.$$

where $r_{c}$ is the roulette constant, which is set to 10 in our experiments.

#### 2.1.2. Image-Stack-Based Calculation Method

**Figure 2:**
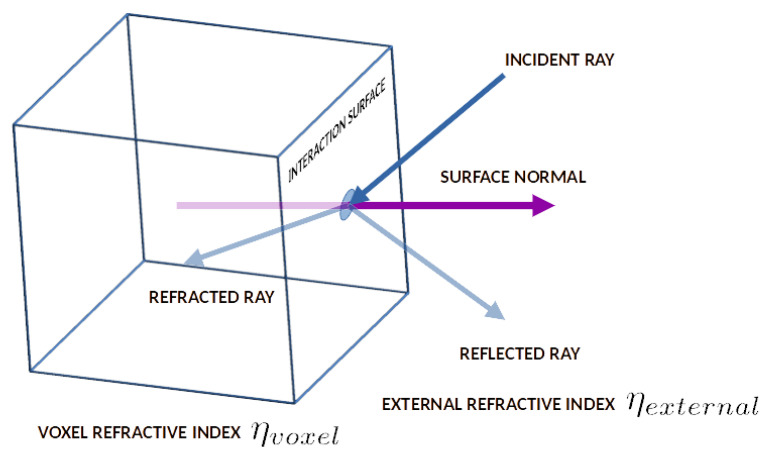
Interactions of the incident ray on a voxel surface.

The Monte Carlo photon transport method described above depends on the position of the photon packet inside the tissue system. The tissues are considered as the axis-aligned cubes consisting of the cube centroid and the cube unit length in the global space. For each tissue boundary detection process, the current tissue material of the photon packet is tracked for changes. If the tissue material changes between any two steps, then the photon is considered to have crossed a tissue boundary. In this scenario, the photon movement is calculated inversely, and a ray casting is performed along the photon packet direction vector on the voxels of the new tissue material. Then, the intersected face of the voxel is computed along with the intersection point and the intersection plane normal in the global space. The photon is then transferred to the intersection point with the estimated photon absorption. From that point, the face normal and photon direction vector are used to calculate the refraction and reflection vectors. The reflectance parameter calculated from Schilick’s approximation is then used to randomly determine the final direction vector of the photon propagation, and the photon position and direction vectors are updated accordingly. Figure 2 shows the photon ray and voxel face interaction cases with the reflection and refraction direction vectors.

#### 2.1.3. Composite Tissue Material Generation

In practice, a voxel-based Monte Carlo photon propagation approach is limited by the memory and computational constraints of the 3D geometry. A tissue with a very thin and curvy surface always produces a large number of voxels, which adequately approximates the light–tissue interaction properties. However, as the number of voxels in the model increases, the simulation system requires more time for each iteration.

To solve this problem, we propose a composite tissue material system in this study. A complex tissue set can be approximated as a lumped set of voxels, and the inner layers can be evaluated programmatically during the runtime. In this manner, the Monte Carlo system does not have to consider computing the high-resolution voxel maps for the ray-casting and boundary detection procedures for the entire model, thereby reducing the computation time required in each step.

**Figure 3:**
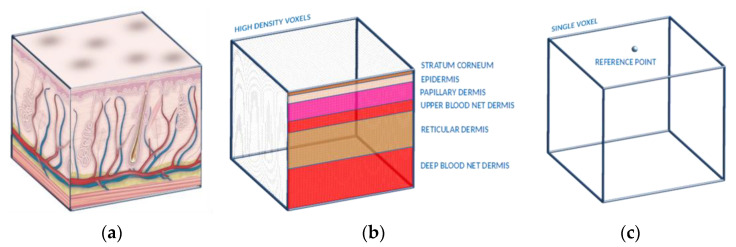
Conceptual illustrations of the (**a**) actual subdermal layers, (**b**) naive voxel generation method (a large number of voxels to faithfully replicate the actual effects of the dermal sublayers), and (**c**) proposed method of composite tissue (in a single voxel, the material properties are calculated during the runtime using the distance of the photon from the reference point(s)).

For example, the dermal sublayers of a skin model can be explained, according to this method. Ideally, the dermis is considered to have about six sublayers, owing to the complex blood net configurations at different depths of the dermis. For the Monte Carlo algorithm implementation, if the dermal sublayers are considered to be different volumes with very high precision bodies, the total number of voxels increases by many times, which exponentially increases the time spent computing each step. In contrast, the dermal layer in our method is lumped as a single set of voxels, and the individual sublayer properties are evaluated by calculating the distance from the vertices of the outer surface of the dermal mesh (outer surface of the stratum corneum sublayer-reference points) during the simulation. Figure 3 shows the composite tissue material generation for the dermal sublayers.

In Figure 3b, the dermal sublayers are shown using the voxel generation method. The different colors in the image indicate different types of tissue. However, in this method, the resolution of the voxels should be very high. In Figure 3c, the same effect can be achieved, but in this way the voxel representation does not need to have a too high resolution.

#### 2.1.4. Time-Resolved Photon Transport

While recording the weight property values of the photon packets, several other properties are also stored, as described earlier. These stored properties are the photon index, global position of the photon packet, photon weight, current tissue material properties, current step, total distance of the photon, and the total elapsed time for the photon packet, among others. The total elapsed time is calculated by adding the time elapsed for a step in the medium to the previously elapsed time for the photon packet. To estimate the elapsed time for each photon packet, the speed of light in that medium is calculated using the refraction index. Finally, to account for the time-resolved data after the simulation, the total simulation time is divided into small temporal bins, and each photon interaction is assessed for the residence in a specific temporal bin.

### 2.2. Model Construction

Models for the Monte Carlo simulation were constructed from 3D MR scans of the wrist by segmenting the image slices according to the different tissue types. The following subsections will discuss these steps in detail.

#### 2.2.1. MR Image Data

**Figure 4:**
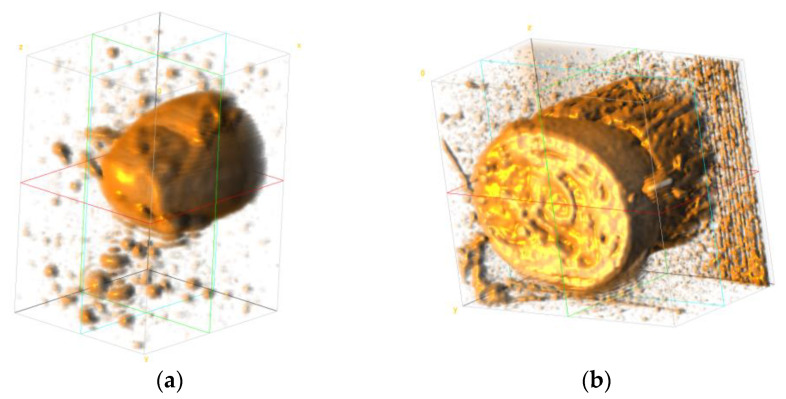
3D MR image data (**a**) fingertip section, (**b**) wrist section.

MR image data of the wrist were obtained from the study by Wang et al. [12]. The dataset contains MRI data of two subjects with 12 poses each. The scanner used in the original study obtained 1 mm thick sample slices with a 0.5 mm overlap. The data were then converted into 0.5 mm isovoxel images. Then, after obtaining the MR data, the images were spatially cropped to select only the region of interest. In our case, only pose 1 (neutral pose) of the male subject was selected for further analysis, since in our implementation, the deformation of the muscular and skeletal tissues does not play a significant role. The regions of interest were the fingertip section of the index finger (from the fingertip to the tip of the middle phalanx) and the wrist section (immediately following the carpals). Figure 4 shows the 3D representation of the two cropped regions from the raw MR data.

#### 2.2.2. Tissue Types

Following the spatially cropping of the MR image scans, the individual slices were manually segmented, based on the tissue types. We considered seven types of tissue materials with the skin having six different sublayers and computed them dynamically during the simulations. The tissue materials are skin, nail, fat, muscle, bone, artery, and vein. The skin tissues are subdivided into the stratum corneum, epidermis, papillary dermis, upper blood net dermis, reticular dermis, and deep blood net dermis. Among the tissue materials, the skin, artery, and vein were considered to have blood content in them. Furthermore, four of the skin sublayers were considered to have blood content, except the stratum corneum and the epidermis. The absorption coefficients of the stratum corneum and epidermis are explained by the following equations [13]:

(16) $$\mu_{a}^{stratcor}\left(\lambda \right)=(\left(0.1-0.3\times 10^{-4}\times \lambda \right)+0.125\times \mu_{a}^{baseline}\left(\lambda \right))\left(1-V_{water}\right)+V_{water}\times \mu_{a}^{water}\left(\lambda \right)$$

(17) $$\mu_{a}^{epidermis}\left(\lambda \right)=(V_{melanin}\times \mu_{a}^{melanin}\left(\lambda \right)+\left(1-V_{melanin}\right)\times \mu_{a}^{baseline}\left(\lambda \right))\left(1-V_{water}\right)+V_{water}\times \mu_{a}^{water}\left(\lambda \right)$$

Here, $V_{water}$, $V_{melanin}$, $\mu_{a}^{baseline}$, and $\mu_{a}^{water}$ indicate the partial volume fractions of water and melanin and the absorption coefficients of the skin baseline and water, respectively. The skin baseline can be expressed in the following form [14];

(18) $$\mu_{a}^{baseline}=7.8375\times 10^{8}\lambda^{-3.48}$$

The remaining sublayers of the skin are generally expressed by the following equation:

(19) $$\mu_{a}^{skinsublayer}=V_{bloodart}\mu_{a}^{art}+V_{bloodvein}\mu_{a}^{vein}+V_{water}\mu_{a}^{water}+\left(1-V_{bloodart}-V_{bloodvein}-V_{water}\right)\mu_{a}^{baseline}$$

where $V_{bloodart}$, $V_{bloodvein}$, $\mu_{a}^{art}$, and $\mu_{a}^{vein}$ represent the partial volume fractions of the arterial and venous blood, as well as the absorption coefficients of the artery and vein, respectively. The volume fraction of each element in a sublayer of the skin and its thickness are shown in Table 1 [15]. To simplify the implementation process, the partial volume fractions of the arterial and venous blood are considered to be equal in each sublayer of the skin.

The absorption coefficients of the arterial and venous blood components of these sublayers are modified depending on the blood volumes of the systolic and diastolic phases of the system. In the systolic phase of the system, the volume fraction of blood is doubled, compared to that of the diastolic phase to simulate the pulsatile nature of the blood flow inside the limb [15]. The arterial and venous blood components in the systolic phase can be expressed by the following equation:

(20) $$\begin{array}{cc} \mu_{a} & =\mu_{a}^{HHb}+P_{HbO}\left(\mu_{a}^{HbO}-\mu_{a}^{HHb}\right)+P_{HbA1c}\left(\mu_{a}^{HbA1c}-\mu_{a}^{HHb}\right)\\ P_{HbO} & =SpO_{2}\left(1-HbA1c\right)\\ P_{HbA1c} & =HbA1c \end{array}$$

In the equation above, $\mu_{a}^{HHb}$, $\mu_{a}^{HbO}$, and $\mu_{a}^{HbA1c}$ indicate the absorption coefficients of deoxyhemoglobin, oxyhemoglobin, and glycated hemoglobin, respectively; $P_{HbO}$ and $P_{HbA1c}$ are the concentration fractions of the respective hemoglobin compounds. Equation (20) can be used to evaluate the absorption coefficients of both the arterial and venous blood. The SpO_2_ value of the venous blood is considered to be 10% below that of the arterial blood [15]. The detailed derivation process of Equation (20) is given in Appendix A.

Although the absorption coefficients of the blood-based elements were modified, the scattering coefficient remained unchanged for a specific wavelength. The anisotropy factor and refraction index values were set to constants, regardless of the wavelength or the systolic and diastolic configurations. The absorption coefficients of muscle [16], bone [17], fat [18], oxy- and deoxyhemoglobin [19], and glycated hemoglobin [20] were obtained from the respective sources. Similarly, the scattering coefficients of muscle [18], bone [18], fat [18], and whole blood [21] were considered from previous studies. The absorption and scattering coefficients of the nail were considered constant for all wavelengths [22]. The absorption and scattering coefficients, the anisotropy factors, and refractive indices are listed in Table 2 for the various tissue components.

#### 2.2.3. Segmentation

**Figure 5:**
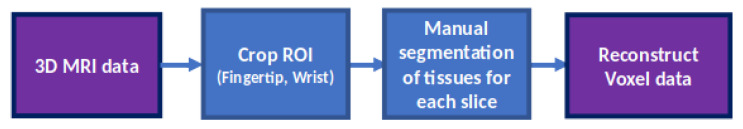
MR image data segmentation process.

The full-wrist MR image slices were first spatially cropped for the regions of interest (i.e., fingertip and wrist sections). These cropped slices were then segmented manually for different tissue materials. We segment the MRI data into seven different tissue types: skin, muscle, fat, bone, artery, vein, and nail. The skin is a composite tissue, which includes six different layers with different optical properties. Following the segmentation, two segmented slices (fingertip and wrist) were reconstructed with 0.5 mm 3D voxel data. This voxel model is used for the Monte Carlo simulations. Figure 5 shows the general procedure of the MR image data segmentation.

##### Fingertip

**Figure 6:**
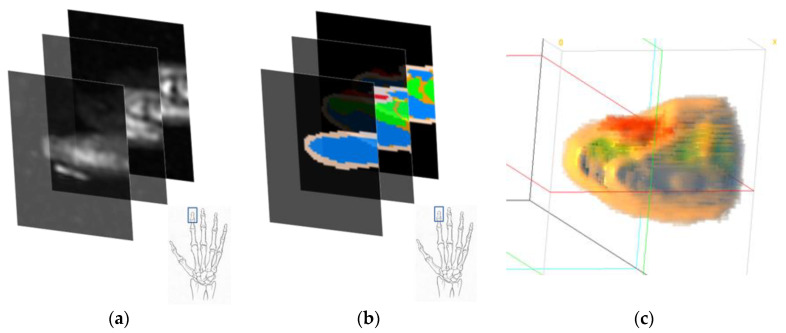
Fingertip (**a**) MR image slices, (**b**) tissue-segmented image slices, and (**c**) reconstructed voxel data from the segmented image slices. Color codes for the skin, muscle, fat, bone, and nail are light coral, orange, blue, green, and red, respectively.

The fingertip MR image slices were segmented for the skin, muscle, fat, bone, and nail. The tendons and ligaments were considered as muscular-type tissues. As the artery and veins in the fingertip region are not pronounced, compared to the other tissues, the arterial and venous blood segments are considered only in the skin tissues. The dimension of the fingertip model was 57 × 76 × 48 voxels. Figure 6 shows the segmented results at different stages.

##### Wrist

**Figure 7:**
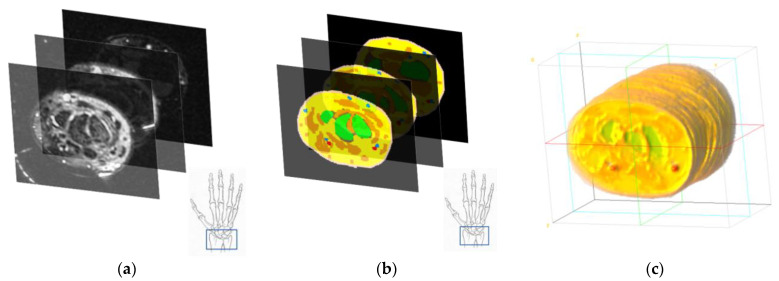
Wrist (**a**) MR image slices, (**b**) tissue-segmented image slices, and (**c**) reconstructed voxel data from the segmented image slices. Color codes for the skin, muscle, fat, bone, artery, and vein are light coral, orange, yellow, green, red, and blue, respectively.

The wrist region of the MR slice was segmented for the skin, muscle, fat, bone, artery, and vein tissues. Similar to the fingertip region segmentation, the wrist region was segmented by considering the ligaments and tendons as muscular-type tissues. Figure 7 shows the segmentation steps of the wrist region.

In both Figure 6 and Figure 7, the MR images were first manually segmented. The different colors in Figure 6b and Figure 7b indicate different tissue types. Following the tissue segmentation, the tissue segmented MR image data was reconstructed as a 3D voxel model.

### 2.3. System Configuration

#### 2.3.1. Source–Receiver Properties

The Monte Carlo simulations are performed by setting the source as a pencil positioned on the soft side of the fingertip and the back side of the wrist (dorsal wrist). The source was configured such that the generated photons have a weight of 1 and 10,000,000 photons are generated from the source position for the simulation in each configuration (specific model, blood volume state (systolic or diastolic), HbA1c, and SpO_2_ values). The receiver (photodetector: PD) properties are not required during the simulation because the results produce 3D voxels of the received intensity data. From these voxel data, a specific position is used to postprocess and simulate the effects of the receiver placement at that position.

#### 2.3.2. Source–Receiver Placement Configurations

**Figure 8:**
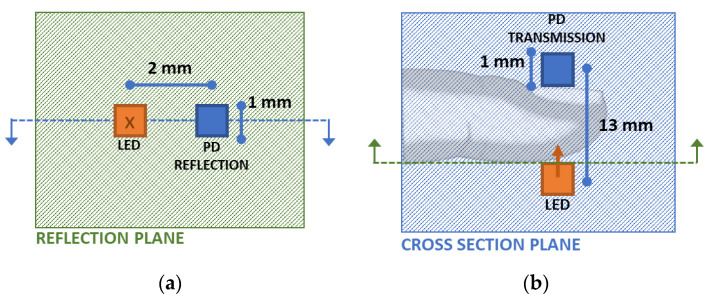
Light-emitting diode (LED) and the photodetector (PD) arrangements for the fingertip model (**a**) on the reflection plane with the blue dotted line and (**b**) on the fingertip cross-sectional plane with the green dotted line indicating the perpendicular planes. The perpendicular planes are indicated with dotted lines and blue-green colors (blue: cross section plane, green: reflection plane).

For the simulation of the fingertip, the transmission- and reflection-type analyses were conducted. Moreover, the wrist model was only considered for reflection-type analysis on the dorsal side. Figure 8 shows the light-emitting diode (LED) and the photodetector (PD) arrangements for the fingertip model. In the fingertip transmission-type analysis, the receiver was placed on the nail after the simulation to calculate the received intensity values at that position. On the contrary, in the fingertip reflection-type analysis, the receiver was placed 2 mm away from the source.

**Figure 9:**
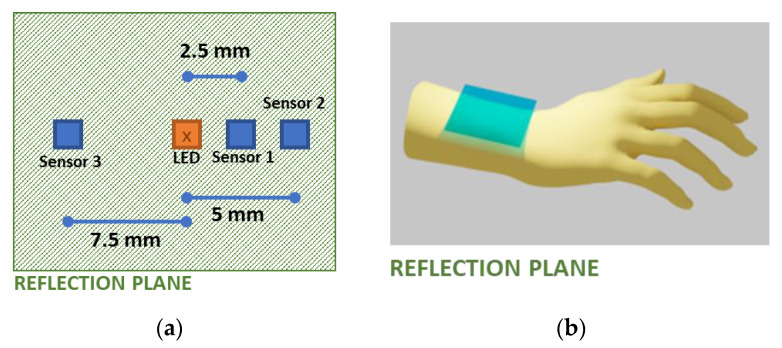
LED-PD arrangements for the wrist model. (**a**) LED-PD arrangement in the reflection plane, (**b**) physical location of the reflection plane for the wrist model.

For analyzing the wrist model, the simulation results are used to assess two different methods. In one method, the received signals at three wavelengths at a 2 mm distance from the source were considered to estimate the HbA1c and SpO_2_ values. In the other method, as shown in Figure 9, a single wavelength of light was selected, and three receivers were placed at three different positions, and from these received intensities, the HbA1c and SpO_2_ values were estimated. The different positions of the receivers in this method were selected by evaluating three different positions with higher variations of the received intensities with respect to the changes in the HbA1c, SpO_2_, and the systolic and diastolic phases. This method is feasible since the 3D reconstructed model has different compositions of materials for different light paths.

Following the configuration of the light sources, the simulations were performed for various HbA1c and SpO_2_ values with the systolic and diastolic phases at three different wavelengths. The SpO_2_ values considered for this study were 0.7, 0.75, 0.8, 0.85, 0.9, 0.95, 0.96, 0.97, 0.98, 0.99, and 1. Similarly, the HbA1c values considered were 0.03, 0.035, 0.04, 0.045, 0.05, 0.055, 0.056, 0.057, 0.058, 0.059, 0.06, 0.061, 0.062, 0.063, 0.064, 0.065, 0.07, 0.075, 0.08, 0.085, 0.09, 0.10, 0.11, 0.12, 0.13, and 0.14. A higher importance was given to the HbA1c and SpO_2_ values that are more common (SpO_2_ from 95% to 100%) or have a clinical importance (HbA1c values from 5.5% to 6.5%).

### 2.4. Calibration

Following the photon transport simulations for the different HbA1c, SpO_2_, and blood volume states, the resulting data consist of 3D voxel intensities of the photons exiting the model at the three different wavelengths (465 nm, 525 nm, 615 nm). From this output intensity voxel data, the data tables are generated for the intensities at specific receiver locations and specific wavelengths of light. The data tables contain the received light intensities at 465 nm, 525 nm, and 615 nm; the molar concentrations of HHb, HbO, and HbA1c; the blood volume phase (systolic or diastolic); and the %HbA1c, and %SpO_2_ values.

The intensity values have different scales than the PPG signals acquired from the fingertip or wrist devices because the source is set to the weight of 1 in the simulation. For this reason, the PPG signals received from the devices and the received intensity values from the Monte Carlo simulations need to be calibrated. To calibrate these two intensity values, a personalized regression model [26] was used (XGBoost regression) including about 4 s of data from each test subject, as these two intensity values should theoretically be related if the noise components have a very low significance. The input to the regression model was the ratios calculated from the experimentally acquired PPG signals at three different wavelengths, as well as 45 temporal- and frequency-domain features of the PPG signals received from the experimental device. The ratios calculated from the received intensities from the Monte Carlo simulations (in short: simulated ratio values) were given as the target of the calibration model. These values reduce the impact of the unknown parameters in a real fingertip or wrist and can provide more dependable values than using light intensities directly from the sensors.

All of the protocols and procedures in this study were approved by the institutional review board (IRB), Kookmin University, Seoul, Korea (approval date: 25 February 2022). The procedures followed the Helsinki Declaration of 1975, as revised in 2008. All human participants agreed in advance to participate and share data for academic research purposes with written informed consent. The IRB protocol number is KMU-202111-BR-286.

These ratios are defined as the ratio of *AC* to *DC* of the two wavelengths of light. The ratio equations for the three wavelengths (465 nm, 525 nm, and 615 nm) are as follows.

(21) $$R_{1}=\frac{{\left[\frac{AC}{DC}\right]}_{525\mathrm{nm}}}{{\left[\frac{AC}{DC}\right]}_{615\mathrm{nm}}}$$

(22) $$R_{2}=\frac{{\left[\frac{AC}{DC}\right]}_{465\mathrm{nm}}}{{\left[\frac{AC}{DC}\right]}_{615\mathrm{nm}}}$$

Moreover, for the wrist model with multiple PDs, the ratios can be constructed by calculating the *AC*/*DC* ratios of any two of the received intensities from the PDs. In our case, the equations can be described as follows:

(23) $$R_{1}=\frac{{\left[\frac{AC}{DC}\right]}_{sensor1}}{{\left[\frac{AC}{DC}\right]}_{sensor3}}$$

(24) $$R_{2}=\frac{{\left[\frac{AC}{DC}\right]}_{sensor2}}{{\left[\frac{AC}{DC}\right]}_{sensor3}}$$

From the two unknowns in the final equations ($P_{HbA1c}$ and $P_{HbO}$) and two signals (either signals from the two wavelengths or signals from two different PDs), we construct a single ratio value using the two ratio equations to solve for the unknown variables. Hence, three signal sources are required.

For training the calibration model, the ratio values of each subject were calculated from the recorded PPG signals. Then, the simulated ratio values were calculated from the simulated data matching the HbA1c and SpO_2_ values of the subject. Then the signal ratio values, 45 features, and the BMI were given as input to the XGBoost calibration model, and the simulated ratio values were set as the target for the model training. The list of features along with the feature equations is provided in Appendix B of this manuscript.

## 3. Results

### 3.1. Simulation Results

Following the Monte Carlo simulations for a total of 572 different HbA1c and SpO_2_ values and the systolic–diastolic phase configurations, we received different intensities of light at the designated receivers for each of the models (fingertip and wrist). The simulation is performed using Equations (1)–(15), where the optical properties of the different tissues are calculated using Equations (16)–(20). Then the ratio values are calculated using Equations (21)–(24).

#### 3.1.1. Fingertip: Transmission-Type

**Figure 10:**
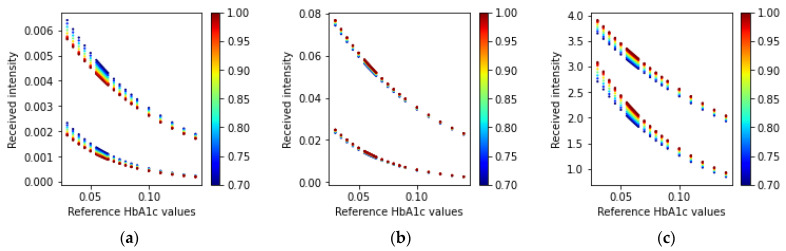
Received intensity values (fingertip: transmission-type) for the three wavelengths: (**a**) 465 nm, (**b**) 525 nm, and (**c**) 615 nm. In each scatter plot, the top adjacent points correspond to the diastolic phase and the bottom points correspond to the systolic phase. The color bars at the side of the plots indicate the different SpO_2_ values ranging from 70% to 100%.

The simulation for the fingertip model and the transmission-type arrangement of the LEDs and PD results in several light intensities for the three wavelengths. Figure 10 illustrates the received intensity values for the three wavelengths of light (465 nm, 525 nm, and 615 nm).

**Figure 11:**
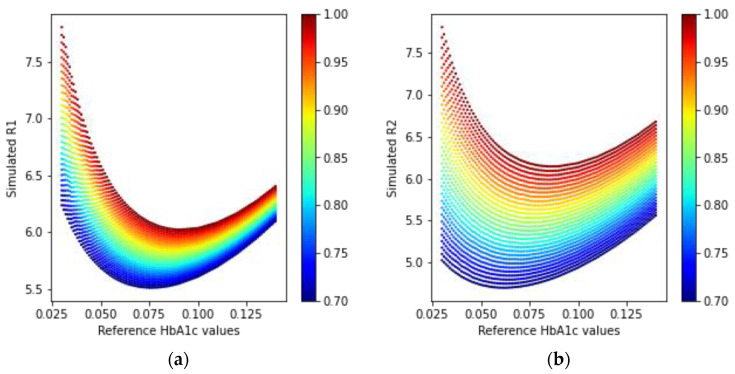
Simulated ratio values (**a**) R1 and (**b**) R2 from the received intensities in the transmission mode PD.

In Figure 10, we can see that the simulated received light intensity is exponentially decreasing as the concentration of HbA1c is increasing. The ratio values calculated using Equations (21) and (22) for the received light in a transmission-type LED and PD arrangement are shown in Figure 11.

The ratio values depicted in Figure 11 have a similar shape in the R-HbA1c space, but different spreads, based on the HbA1c values in the simulated media. Using these two ratio values, it is possible to model the estimation of HbA1c.

#### 3.1.2. Fingertip: Reflection-Type

**Figure 12:**
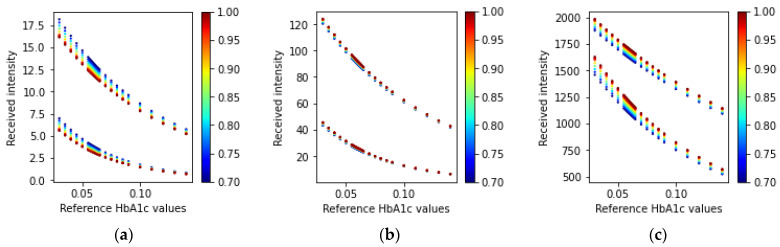
Received intensity values (fingertip: reflection-type) for the three wavelengths (**a**) 465 nm, (**b**) 525 nm, and (**c**) 615 nm. In each scatter plot, the top adjacent points correspond to the diastolic phase and the bottom points correspond to the systolic phase. The color bars at the side of the plots indicate the various SpO_2_ values ranging from 70% to 100%.

Similar to the transmission-type simulation of the fingertip model, the reflection-type simulations result in received intensities at three different wavelengths. Figure 12 shows the received intensities for the simulation of the reflection-type arrangement of the fingertip model.

**Figure 13:**
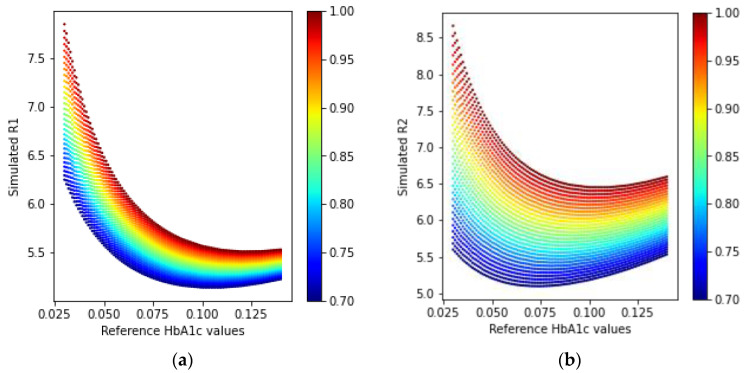
Simulated ratio values (**a**) R1 and (**b**) R2 from the received intensities in the reflection-type arrangement.

Similarly, the simulated ratio values using Equations (21) and (22) for the reflection-type arrangement of the LEDs and PD are shown in Figure 13.

A similar trend in the received simulated intensity as in the transmission-type can also be seen in Figure 12. In Figure 13, the reflected ratio values can also be modeled to estimate HbA1c since these have different spreads in the R-HbA1c space.

#### 3.1.3. Wrist: One PD and Multiple Wavelength LEDs

**Figure 14:**
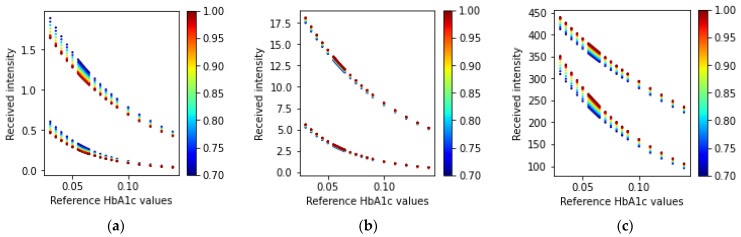
Received intensity values for the three wavelengths (**a**) 465 nm, (**b**) 525 nm, and (**c**) 615 nm. In each scatter plot, the top adjacent points correspond to the diastolic phase and the bottom points correspond to the systolic phase. The color bars at the side of the plots indicate the different SpO_2_ values ranging from 70% to 100%.

For the wrist model, sensor 1, shown in Figure 9, receives three wavelengths of light. The received intensities are illustrated in Figure 14 corresponding to different HbA1c and SpO_2_ values.

**Figure 15:**
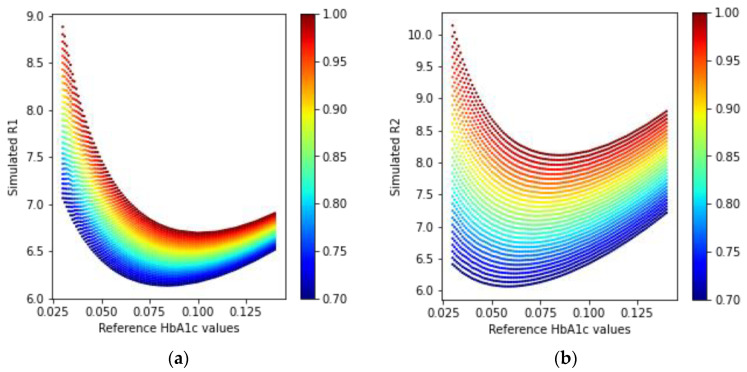
Simulated ratio values (**a**) R1 and (**b**) R2 from the received intensities in the one PD and multiple wavelength LEDs arrangement of the wrist model. The color bars at the side of the plots indicate the different SpO_2_ values ranging from 70% to 100%.

The ratio values found from the Monte Carlo simulations with one PD and multiple LEDs are shown in Figure 15.

#### 3.1.4. Wrist: Multiple PDs and One Wavelength LED

**Figure 16:**
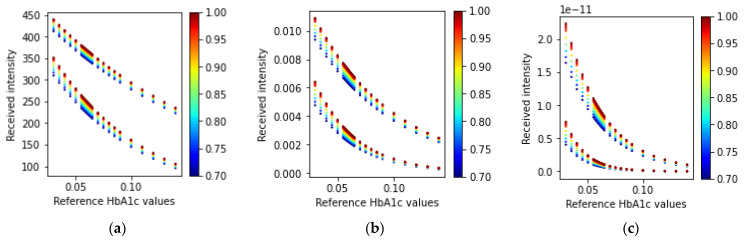
Received intensity values at the three different PDs shown in Figure 9 (**a**) sensor 1, (**b**) sensor 2, and (**c**) sensor 3 for the 615 nm wavelength of light. In each scatter plot, the top adjacent points correspond to the diastolic phase and the bottom points correspond to the systolic phase. The color bars at the side of the plots indicate the different SpO_2_ values ranging from 70% to 100%.

For the three PDs and the single LED arrangement in the wrist model, the PDs are placed at different distances from the LED, as described in Section 2.3.2 “Source–receiver placement configurations”. This procedure creates different paths for the photon packets, and hence three independent solutions of the same model can be generated. These solutions can be used to calculate the two ratio values in Equations (21) and (22). For this purpose, a wavelength with a higher penetration depth (615 nm) was chosen as having a higher penetration depth that will result in a higher received intensity at the farthest PD from the LED location. The light intensities received at the different PDs for an LED with a wavelength of 615 nm are shown in Figure 16.

**Figure 17:**
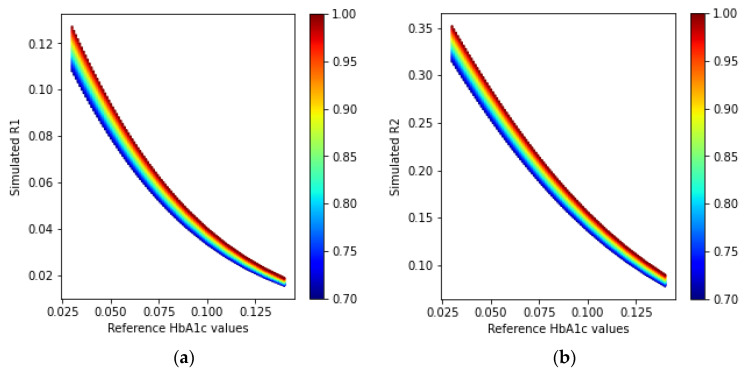
Simulated ratio values (**a**) R1 and (**b**) R2 from the received intensities in the multiple PDs and one wavelength LED arrangement in the wrist model. The color bars at the side of the plots indicate the different SpO_2_ values ranging from 70% to 100%.

The ratio values calculated using Equations (23) and (24) for the multiple PDs with a single-wavelength LED arrangement are illustrated in Figure 17.

Comparing Figure 15 and Figure 17, we can state that the multiple PDs and one LED method has a unique spread in the R-HbA1c space. Similar to the previous implementations, this information can also be used to construct a model to estimate the HbA1c levels.

### 3.2. Human Data Demographics

All of the data for evaluating the experimental results were acquired from real subjects under the supervision of the institutional review board (IRB) of Kookmin University, Seoul, Korea. For the analysis of the fingertip model results, the data from 30 subjects were acquired with consent to use the recorded data for research purposes. To evaluate a wrist model with multiple LEDs and a single PD, 28 persons consented to provide their PPG data. For all recordings, fingertip and wrist PPG signal acquisition devices use the TCS34725 sensor with white LEDs. The white LEDs used in this study are of the phosphor-blue type. The fingertip reflection-type device contains four low-power white LEDs and one PD on the same side of the finger. The fingertip transmission-type device contains one high-power white LED on one side and a PD on the other side of the fingertip. The wrist device contains only four low-power white LEDs and one PD, similar to the fingertip reflection-type sensor. The sensor records the intensities at three wavelengths (465 nm, 525 nm, and 615 nm) simultaneously. We have taken measures to make the data free from environmental influences as much as possible (usage of a clip type device for the fingertip and using optical barriers to block light leaking into the sensor for both the fingertip and wrist type devices). To record the reference HbA1c and SpO_2_ values, we used the BioHermes A1c EZ and Schiller Argus OXM Plus devices, respectively. The demographics of the acquired data (for fingertip and wrist) are given in Table 3.

The fingertip recordings included 4 min of data per subject, where 2 min were for the transmission-type PPG and the remaining 2 min were for the reflection-type PPG signals. Moreover, the wrist PPG signal included only the reflection-type PPG data for an average of 2 min.

### 3.3. Model Validation Results

Next, after obtaining the simulation results and calculating the simulated ratios, the ratio values found from the recorded PPG signals were calibrated and analyzed for evaluating the model performance. Then, Equation (20) was used to calculate the HbA1c and SpO_2_ values from the definition of $P_{HbA1c}$ and $P_{HbO}$ values reported in Equation (20).

#### 3.3.1. Fingertip: Transmission-Type

**Figure 18:**
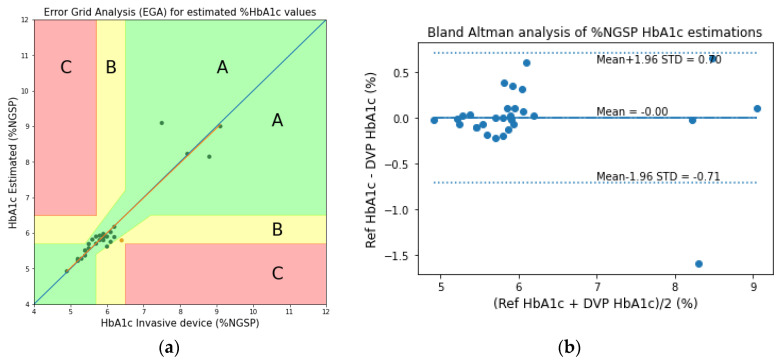
(**a**) Error grid analysis (EGA) and (**b**) the Bland–Altman analysis plots of the experimentally acquired data for the transmission-type signal of the fingertip model.

By analyzing the estimated HbA1c values of the transmission-type PPG signals for the fingertip model, the error grid analysis (EGA) and the Bland–Altman analysis plots are shown in Figure 18. In the EGA, Zone A represents the values within 20% of the reference sensor. Zone B contains points that are outside of 20% but would not lead to inappropriate treatment. Zone C are those points leading to unnecessary treatment. From the Bland–Altman analysis, the bias is found to be −0.003 ± 0.36.

The estimated HbA1c values are evaluated using several metrics. These are the mean-squared error (MSE), mean error (ME), mean absolute deviation (MAD), root mean-squared error (RMSE), and Pearson’s r. The evaluation metrics for the transmission-type fingertip model are given in Table 4.

**Figure 19:**
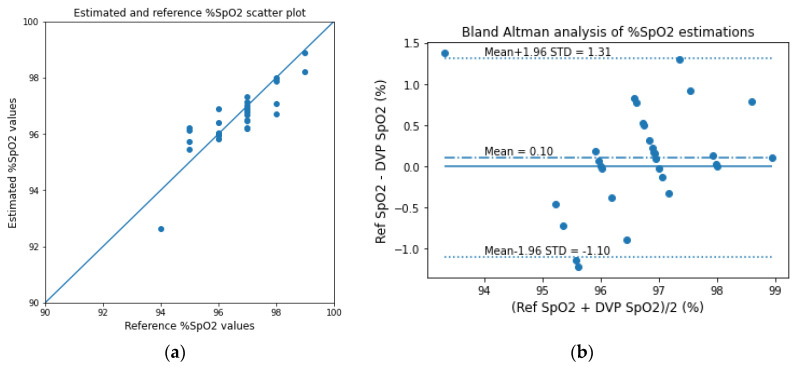
(**a**) Scatter plot for the reference and estimated SpO_2_ values and (**b**) the Bland–Altman analysis plots of the experimentally acquired data for the transmission-type signal of the fingertip model.

The estimated SpO_2_ values are also evaluated as scatter and the Bland–Altman analysis plots in Figure 19, and the corresponding evaluation metrics are given in Table 5. For evaluating the SpO_2_ values, we use the reference closeness factor (RCF) instead of Pearson’s r for a better assessment of the small-range data. The RCF can be defined as follows:

(25) $$RCF=\frac{1}{N}\overset{N}{\underset{i=1}{\sum }}\left(1-\frac{SpO_{2}^{ref}\left(i\right)-SpO_{2}^{est}\left(i\right)}{100}\right)$$

The bias of the Bland–Altman analysis for the SpO_2_ evaluation was found to be 0.10 ± 0.62 for the transmission-type fingertip model.

#### 3.3.2. Fingertip: Reflection-Type

**Figure 20:**
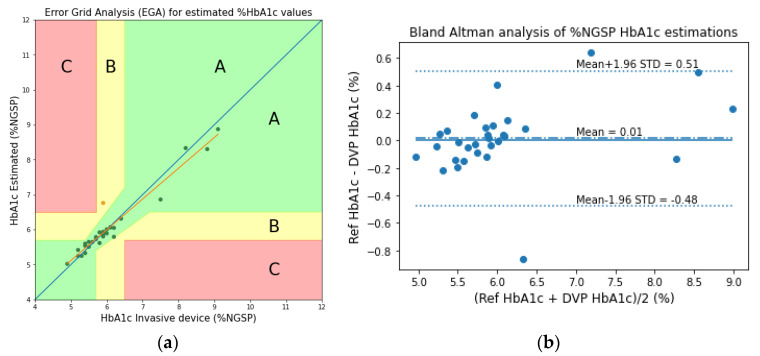
(**a**) EGA and (**b**) the Bland–Altman analysis plots of the experimentally acquired data for the reflection-type signal of the fingertip model.

**Figure 21:**
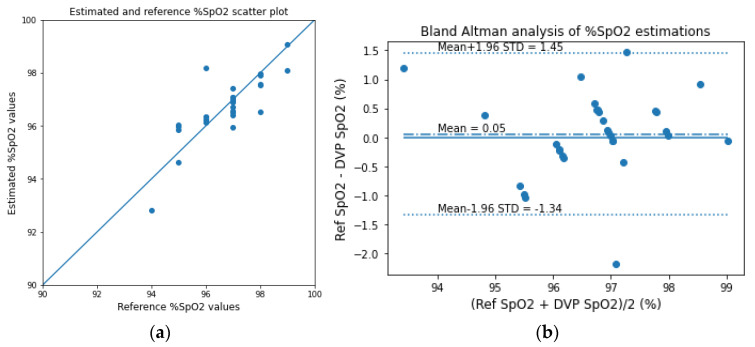
(**a**) Scatter plot for the reference and estimated SpO_2_ values and (**b**) the Bland–Altman analysis plots of the experimentally acquired data for the reflection-type signal of the fingertip model.

Similar to the analyses of the transmission-type fingertip models, the EGA and Bland–Altman analysis plots of HbA1c are shown in Figure 20, and the scatter and Bland–Altman analysis plots of SpO_2_ are shown in Figure 21 for the reflection-type fingertip model. From the Bland–Altman analysis plots, the biases were found to be 0.01 ± 0.25 and 0.05 ± 0.71 for the HbA1c and SpO_2_ estimations, respectively. The evaluation metrics for HbA1c and SpO_2_ are given in Table 6 and Table 7, respectively.

#### 3.3.3. Wrist: One PD and Multiple Wavelength LEDs

**Figure 22:**
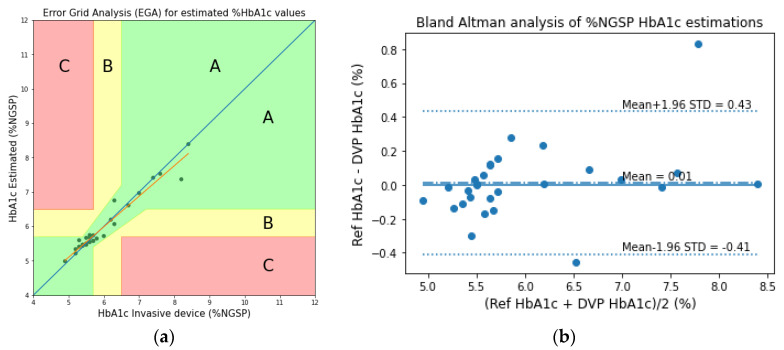
(**a**) EGA and (**b**) the Bland–Altman analysis plots of the experimentally acquired data for the single PD reflection-type signal of the wrist model.

**Figure 23:**
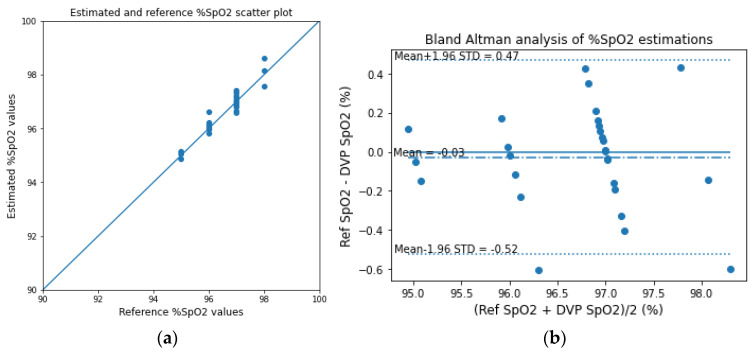
(**a**) Scatter plot for the reference and estimated SpO_2_ values and (**b**) the Bland–Altman analysis plot of the experimentally acquired data for the single PD reflection-type signal of the wrist model.

For analyzing the one PD and multiple (three) wavelength LED process, Figure 22 shows the EGA and the Bland–Altman analysis plots (bias 0.01 ± 0.21) for the HbA1c estimations. Figure 23 shows the SpO_2_ estimation scatter and the Bland–Altman analysis plots (bias −0.03 ± 0.26). Table 8 and Table 9 show the evaluation metrics for the HbA1c and SpO_2_ estimations, respectively.

### 3.4. Results Comparison

We compared the results of the current study with those of previous studies on the noninvasive estimation of the HbA1c level in vivo. One of the previous studies used the simple Beer–Lambert-law-based model to estimate the HbA1c level in vivo [10]. Another work focused on the estimations, based on the photon diffusion theorem by considering both the transmission- and reflection-type PPG signals [26]. Table 10 shows the comparison of the previous and present studies for the fingertip system.

## 4. Discussion

From the simulated results, it is seen that the fingertip transmission- and reflection-type models yield simulated ratios that are not visually differentiable. However, the simulated R2 of the fingertip reflection-type model has a higher range of ratios for a specific value of HbA1c. This indicates that HbA1c is less sensitive to the ratio values and can provide a more stable output for the same amount of noise, as compared to the fingertip transmission-type model. A similar phenomenon is observed for the wrist model with the single PD. The model has a much higher range of ratio values for a specific HbA1c value; hence, the resulting HbA1c and SpO_2_ values are more accurate than those in the previous two models.

The wrist model with multiple PDs shows a very different trend. Although we show a promising method to estimate the HbA1c and SpO_2_ values using only one wavelength from the relationship between the HbA1c, SpO_2_, and ratio values, as shown in Figure 17, this process may produce sensitivity issues at present owing to the narrow range of ratios for a specific HbA1c value. The effects of the sensitivity are reflected in the results from the experimental studies from different human subjects. The fingertip transmission model shows the lowest Pearson r value (0.94), whereas the values of the fingertip and wrist reflection models with a single PD are almost similar (0.96 and 0.97, respectively). However, for the wrist model with a single PD, the bias and limits of agreement from the Bland–Altman analyses are seen to be lower, compared to those of the fingertip reflection model, owing to the higher spread of the ratio values, as seen in Figure 15.

Furthermore, compared to various noninvasive state-of-the-art processes of estimating HbA1c, it can be seen that our fingertip reflection model performs the best, compared to other implementations. Here, we should note that, the previous studies depend on many different signals and physiological features to estimate the HbA1c and blood oxygenation levels. Only the proposed method shows a definitive pathway to minimize the dependency on physiological parameters and uses a small set of features to estimate the physiological parameters. Here, the use of a reduced set of physiological features does not affect the estimated values in the proposed methods. From the results, we can see that despite having a lower number of features, our Monte Carlo based methods perform better than the other methods. This is possible because the proposed method uses a more accurate MRI based 3D model and accurate heterogeneous composition of different tissue types.

Moreover, the proposed model can be implemented in the embedded hardware with a high degree of optimization and with minimal use of computing resources. Although this proposed method does not include the power optimization of LEDs and PDs, the design of this algorithm provides a design flexibility with extremely minimal power usage by requiring only a moderate sampling rate per signal to identify the properties of a PPG signal. We want to affirm that the minimum requirements of a PPG signal are very basic and our method uses a very low power in the side of the operating LEDs and PDs. Moreover, the one LED and multiple PDs method described here can further minimize the power usage, since the LED is the most power-consuming component inside a PPG-based device, followed by the processing unit.

Although this work provides a comprehensive and accurate method to estimate the noninvasive HbA1c values in vivo, this may also have some potential limitations, which can be further studied in future studies. There are a number of parameters considered during the design of the geometric model from the MR image data. However, complex biological media may contain many unknown parameters which can affect the accuracy of the HbA1c estimation. We are also actively working to mitigate the various dermal properties among different persons, to make the models more robust in different scenarios.

## 5. Conclusions

In this study, we developed a method of a noninvasive estimation of the HbA1c and SpO_2_ values using the Monte Carlo process. In this proposed method, we utilized 3D MR image scan data to extract tissue information from specific regions of interest (fingertip and wrist sections). Then, the tissues were voxelized and segmented with assigned bio-optical properties. Four different LED–PD arrangements were proposed for the fingertip and wrist models. Among these arrangements, the fingertip transmission, fingertip reflection, and wrist model with a single PD were tested and validated with the experimental data collected from human subjects. The results from the experimentally acquired data show very high correlations with the simulated results. A comparison of the fingertip models of this proposed method with the previously studied methods shows significant improvements in the estimation accuracy of HbA1c as well as SpO_2_. Furthermore, the proposed wrist model with multiple LEDs and a single PD exhibited the highest correlation with the reference experimental data among the three tested models. The implementation of this method on the wrist can enable the design of smartwatch devices with the capability to diagnose diabetes and estimate the glycated hemoglobin (HbA1c) in vivo, in real time, noninvasively. This can be a game-changer technology helping diabetic and health-conscious people. In the future, we will focus more on the changes of the skin parameters for different persons to produce more accurate results.

Moreover, the proposed method with multiple PDs and one-wavelength LED promises to simplify the circuit design process owing to the use of a single LED to estimate the HbA1c level in vivo. As the usage of a single LED vastly reduces the complexity of the LED control circuitry, during a wearable device implementation, this method of noninvasive in vivo estimation of HbA1c and SpO_2_ can be a suitable substitute for invasive state-of-the-art methods.

## Figures and Tables

**Table 1:** Fractional volume and layer properties of the different skin sublayers.

Skin Sublayer Name	$V_{bloodart}$	$V_{bloodvein}$	$V_{water}$	$V_{melanin}$	Layer Thickness [mm]
Stratum corneum	0	0	0.05	0	0.02
Epidermis	0	0	0.2	0.1	0.25
Papillary dermis	0.02	0.02	0.5	0	0.1
Upper blood net dermis	0.15	0.15	0.6	0	0.08
Reticular dermis	0.02	0.02	0.7	0	0.2
Deep blood net dermis	0.05	0.05	0.05	0	0.3

**Table 2:** Optical properties of the biomaterials at three wavelengths (465 nm, 525 nm, and 615 nm).

Material	Absorption Coefficient $\mu_{a}$ $\;[mm^{-1}]$			Scattering Coefficient $\mu_{s}$ $\;[mm^{-1}]$			Anisotropy Factor g	Refractive Index η
	465 nm	525 nm	615 nm	465 nm	525 nm	615 nm		
Oxyhemoglobin	8.94	7.18	0.27	-			-	-
Deoxyhemoglobin	4.35	8.18	1.76	-			-	-
Glycated hemoglobin	127.68	105.85	39.66	-			-	-
Whole blood	-			84.61	59.17	53.00	0.995 [23]	1.354 [24]
Melanin	88.66	58.12	33.51	-			-	-
Skin baseline	0.163	0.110	0.066	-			-	-
Muscle	0.88	1.17	0.22	2.41	1.71	1.09	0.5 [15]	1.37 [25]
Fat	0.005	0.001	0.0004	6.47	5.96	5.35	0.75 [13]	1.44 [25]
Bone	0.118	0.118	0.068	53.40	44.68	35.41	0.92 [15]	1.37 [17]
Nail	0.012			21			0.90 [22]	1.51 [22]

**Table 3:** Dataset demographics.

Dataset	HbA1c (%)			SpO_2_ (%)			Age (Mean ± SD)	BMI (Mean ± SD)
	Min	Max	Mean ± SD	Min	Max	Mean ± SD		
Fingertip	4.9	9.1	6.08 ± 0.99	94	99	96.74 ± 1.16	34.6 ± 13.01	27.87 ± 4.05
Wrist	4.9	8.4	6.01 ± 0.90	95	98	96.68 ± 0.80	42.67 ± 15.11	25.60 ± 4.15

**Table 4:** Evaluation metrics of HbA1c for the transmission-type fingertip model.

MSE	ME	MAD	RMSE	Pearson’s r
0.13	−0.003	0.19	0.36	0.94

**Table 5:** Evaluation metrics of SpO_2_ for the transmission-type fingertip model.

MSE	ME	MAD	RMSE	RCF
0.39	0.10	0.46	0.62	0.9953

**Table 6:** Evaluation metrics of HbA1c for the reflection-type fingertip model.

MSE	ME	MAD	RMSE	Pearson’s r
0.06	0.01	0.16	0.25	0.96

**Table 7:** Evaluation metrics of SpO_2_ for the reflection-type fingertip model.

MSE	ME	MAD	RMSE	RCF
0.51	0.05	0.51	0.71	0.9948

**Table 8:** Evaluation metrics of HbA1c for the single PD reflection-type wrist model.

MSE	ME	MAD	RMSE	Pearson’s r
0.05	0.01	0.13	0.21	0.97

**Table 9:** Evaluation metrics of SpO_2_ for the single PD reflection-type wrist model.

MSE	ME	MAD	RMSE	RCF
0.06	−0.03	0.19	0.25	0.9981

**Table 10:** Comparison of the different methods for estimating HbA1c in vivo, noninvasively.

Method	HbA1c Pearson’s r	SpO_2_ RCF
Beer–Lambert fingertip blood-vessel model [10]	0.90	0.988
Beer–Lambert fingertip whole-finger model [10]	0.95	0.986
Photon diffusion fingertip reflection model [26]	0.91	0.988
Photon diffusion fingertip transmission model [26]	0.89	0.987
Proposed Monte Carlo fingertip reflection model	**0.96**	**0.995**
Proposed Monte Carlo fingertip transmission model	0.94	**0.995**

The boldface text indicates the best results.

**Figure A1:**
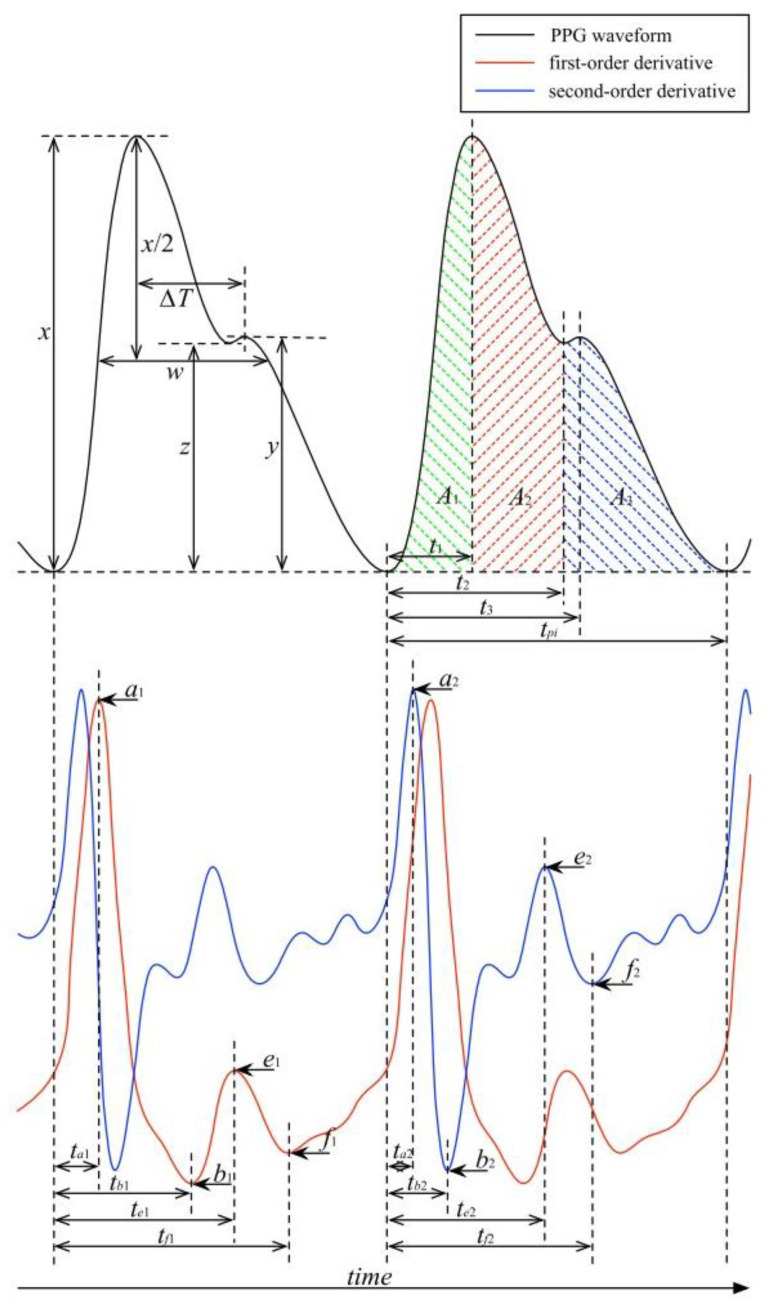
Considered feature points on the PPG pulse waveform and its corresponding first-order and second-order derivatives. All credit goes to the original author of the image [27].

## Data Availability

The dataset used in this research is available upon a valid request to any of the authors of this research article.

## Appendix A

### $\mu_{a}$ Derivation Process

$$\mu_{a}=\mu_{a}^{HHb}+P_{HbO}\left(\mu_{a}^{HbO}-\mu_{a}^{HHb}\right)+P_{HbA1c}\left(\mu_{a}^{HbA1c}-\mu_{a}^{HHb}\right)$$

The absorption coefficient of the arterial or venous blood can be stated as follows.

(A1) $$\mu_{a}=\epsilon_{a}^{HHb}c^{HHb}+\epsilon_{a}^{HbO}c^{HbO}+\epsilon_{a}^{HbA1c}c^{HbA1c}$$

Here, the ϵ and c indicate the molar absorption coefficient and the molar concentration of the different elements. Now let:

(A2) $$P_{HbO}=\frac{c^{HbO}}{c^{HHb}+c^{HbO}+c^{HbA1c}}=\frac{c^{HbO}}{c^{art}}$$

(A3) $$P_{HbA1c}=\frac{c^{HbA1c}}{c^{HHb}+c^{HbO}+c^{HbA1c}}=\frac{c^{HbA1c}}{c^{art}}$$

So:

(A4) $$P_{HHb}=1-P_{HbO}-P_{HbA1c}$$

Now from (A1):

$$\mu_{a}=c^{art}\left(\frac{\epsilon_{a}^{HHb}c^{HHb}}{c^{art}}+\frac{\epsilon_{a}^{HbO}c^{HbO}}{c^{art}}+\frac{\epsilon_{a}^{HbA1c}c^{HbA1c}}{c^{art}}\right)$$

Replacing the $P_{HbO}$, $P_{HHb}$, and $P_{HbA1c}$ terms from (A2)–(A4), we obtain:

(A5) $$\mu_{a}=c^{art}\left(\epsilon_{a}^{HHb}P_{HHb}+\epsilon_{a}^{HbO}P_{HbO}+\epsilon_{a}^{HbA1c}P_{HbA1c}\right)$$

Now let:

(A6) $$\mu_{a}^{HbA1c}=c^{art}\epsilon_{a}^{HbA1c}$$

(A7) $$\mu_{a}^{HbO}=c^{art}\epsilon_{a}^{HbO}$$

(A8) $$\mu_{a}^{HHb}=c^{art}\epsilon_{a}^{HHb}$$

So, applying (A6)–(A8) in (A5), we obtain the final equation:

$$\mu_{a}=\mu_{a}^{HHb}+P_{HbO}\left(\mu_{a}^{HbO}-\mu_{a}^{HHb}\right)+P_{HbA1c}\left(\mu_{a}^{HbA1c}-\mu_{a}^{HHb}\right)$$

## Appendix B

### Features

**Table A1:** Table of the features used to calibrate the models.

Serial	Feature Description	Feature
1	Systolic peak, x	
2	Diastolic peak, y	
3	Dicrotic notch, z	
4	Pulse interval, $t_{pi}$	
5	Augmentation index	y/x
6	Relative augmentation index	$\left(x-y\right)/y$
7		z/x
8		$\left(y-z\right)/x$
9	Systolic peak time, $t_{1}$	
10	Dicrotic notch time, $t_{2}$	
11	Diastolic peak time, $t_{3}$	
12	Time between dicrotic notch and diastolic peak, Δt	$t_{3}-t_{2}$
13	Time between half systolic peak points, w	
14	Inflection point area ratio, $A_{2}/A_{1}$	
15	Systolic peak rising slope	$t_{1}/x$
16	Diastolic peak falling slope	$y/\left(t_{pi}-t_{3}\right)$
17		$t_{1}/t_{pi}$
18		$t_{2}/t_{pi}$
19		$t_{3}/t_{pi}$
20		$\Delta t/t_{pi}$
21		$t_{a1}$
22		$t_{b1}$
23		$t_{e1}$
24		$t_{f1}$
25		$b_{2}/a_{2}$
26		$e_{2}/a_{2}$
27		$\left(b_{2}+e_{2}\right)/a_{2}$
28		$t_{a2}$
29		$t_{b2}$
30		$t_{a1}/t_{pi}$
31		$t_{b1}/t_{pi}$
32		$t_{e1}/t_{pi}$
33		$t_{f1}/t_{pi}$
34		$t_{a2}/t_{pi}$
35		$t_{b2}/t_{pi}$
36		$\left(t_{a1}+t_{a2}\right)/t_{pi}$
37		$\left(t_{b1}+t_{b2}\right)/t_{pi}$
38		$\left(t_{e1}+t_{2}\right)/t_{pi}$
39		$\left(t_{f1}+t_{3}\right)/t_{pi}$
40	Fundamental component frequency	$f_{base}$
41	Fundamental component magnitude	$\left|s_{base}\right|$
42	2nd harmonic frequency	$f_{2}$
43	2nd harmonic magnitude	$\left|s_{2}\right|$
44	3rd harmonic frequency	$f_{3}$
45	3rd harmonic magnitude	$\left|s_{3}\right|$
